# Insights on eating behaviors among adult females in Jordan: A national survey study

**DOI:** 10.1097/MD.0000000000044073

**Published:** 2025-08-22

**Authors:** Kanar Sweiss, Abdallah Y. Naser, Alaa A. Alsharif

**Affiliations:** a Department of Basic Pharmaceutical Sciences, Faculty of Pharmacy, Middle East University (MEU), Amman, Jordan; b Department of Applied Pharmaceutical Sciences and Clinical Pharmacy, Faculty of Pharmacy, Isra University, Amman, Jordan; c Department of Pharmacy Practice, College of Pharmacy, Princess Nourah bint Abdulrahman University, Riyadh, Saudi Arabia.

**Keywords:** approach, avoidance, behavior, eating, females, food

## Abstract

Culture plays an important role in determining the type of food in societies. Most people suffer from challenges related to eating habits as many young women have a lack of acceptance of their body shape. Between April 20, 2025 and May 31, 2025, a cross-sectional study was conducted utilizing an online survey tool in Jordan to examine female adults eating behavior. This study used the Arabic version of a previously validated questionnaire named Adult Eating Behavior Questionnaire. Logistic regression analysis was conducted to predict significant factors influencing the food behaviors. A total of 702 females were involved in this study. The mean food approach subscale score was 44.8 ± 10.3, demonstrating more positive attitude towards approaching foods. Among the food approach subscales, “Enjoyment of Food” showed the highest mean score (21.23 ± 4.12), corresponding to 70.7% of the maximum possible score, while “Emotional Over-Eating” had the lowest mean score at 12.64 ± 5.6 (50.5%). The mean food avoidance subscale score was 38.1 ± 6.2, demonstrating more positive attitude towards foods avoidance. In the food avoidance subscales, “Food Fussiness” recorded the highest mean score (14.34 ± 2.36; 71.7%), while “slowness in Eating” had the lowest mean score at 11.55 ± 3.87 (57.7%). Overweight and obese individuals had higher odds of food approach behaviors compared to underweight individuals (OR = 1.77, 95% CI: 1.09–2.88, *P* = .02, OR = 3.40, 95% CI: 1.41–8.19, *P* = .006), respectively. Conversely, overweight and obese participants had significantly lower odds of food avoidance behaviors (OR = 0.38, 95% CI: 0.23–0.62, *P* < .001, OR = 0.27, 95% CI: 0.11–0.67, *P* = .00), respectively. Additionally, participants working in nonmedical fields had lower odds of food avoidance behaviors compared to those not working (OR = 0.36, 95% CI: 0.16–0.78, *P* = .01). Both food approach and avoidance behaviors were positively viewed by females in Jordan, with “Food Fussiness” and “Enjoyment of Food” being the most prominent features. Overweight and obese females had higher food approach and lower food avoidance behaviors, suggesting a behavioral pattern that may contribute to excess weight. These findings highlight the complex link between sociodemographic parameters including body weight and diet.

## 1. Introduction

Eating pattern is a style that followed by individuals related to their food preferences and choices. The persons can choose their meal types and how much the quantity they would consume. In additions to that, the behaviors toward food are affected by the culture and society.^[1]^ On the other hand, the eating habits are not only including the process of eating and chewing food, but they also start from going into shopping center and select substances, prepare, and cook them, and the food that consumed by individual is referred to diet routine.^[2]^ Furthermore, healthy diet can reduce the risk of diseases, this diet includes eating high amounts of some kind of food such as vegetables, fruits, fibers, and proteins and protect the body from harmful ingredients such as saturated oils and fats.^[3]^ Maintaining the daily diet is important for the human health, so the persons must control their food intake to avoid the complications such as obesity or thinness which may lead to many health issues in heart, kidneys, endocrine, and immunity systems.^[4–7]^ When comparing men and women, previous literature fond that nutritional needs differ according to gender, as women have special nutritional requirements due to hormonal changes they go through, such as menstruation, pregnancy, breastfeeding, etc. Women tend to store more fat than men, so the rate of obesity among them is higher compared to men. Also, some diseases related to food, such as loss of appetite or bulimia, increase. Therefore, adhering to them to a balanced diet is considered a very important matter to maintain health, as eating food that contains some nutritional supplements such as calcium and vitamin D helps to support bone health. A balanced diet also maintains sexual health in women, avoids some problems related to obesity, improves mental health, and avoids some chronic diseases.^[8]^ Another thing that affects eating habits is the common societal customs. For example, when an individual is part of a group, the group imposes the opinion of the majority. Therefore, the eating behaviors that must be followed are determined, especially among individuals who are socially connected.^[9–13]^ On the other hand, culture plays an important role in determining the type of food in societies, because culture is affected by some factors such as the type of food that is grown, transportation and distribution mechanisms, the financial level of families and individuals in society, and how food is prepared. Therefore, some societies practice some patterns and practices specific to their culture, as they buy some foods that can be repeated in these societies and become a habit for individuals.^[14]^ When we are talking about eating habits, most people suffer from some challenges, the most important of which is not accepting their body shape, as many young women have a lack of acceptance of their body shape due to the spread of ideal bodies through social media, which affects the mental and psychological health of these young women.^[15–19]^ Another challenge is the diets that are widespread on social media, which can negatively affect the minds of young women. They sometimes encourage acceptance of the body as it is and therefore advise young women to eat without paying attention to nutritional values. On the other hand, they can sometimes convey the image that a slim body is better, so it advises eating small amounts of food, which negatively affects the health of young women’s bodies.^[20]^ Hormonal changes also have a control on the appetite, as estrogen for example decreases the food consumption.^[21]^ Eating problems occupy a high percentage in the world, as studies have shown that there is an increase in problems related to eating habits among citizens.^[22]^ It has been found that there is a relationship between a person’s demographic factors and eating disorders, it has been noted that people with low income tend to consume less useful food than people with high income,^[23]^ also the social media has a greater impact on the food decisions among people.^[18,24]^ Paying attention to eating is one of the important things that must be taken into consideration and given a great deal of study because it is linked to many diseases.

A previous study examined adult eating behavior in Saudi Arabia and found that slowness in eating (SE) was negatively associated with BMI, while emotional overeating (EOE) was positively associated.^[25]^ A previous review article that examined eating disorders in the Arab world found that females were most at risk for eating disorder symptoms compared to males.^[26]^ Another systematic review found that certain eating behaviors might be related to peripartum weight change.^[27]^ Another systematic review and meta-analysis found that tendencies toward healthy eating habits are comparable across males and females, however, pathologically healthful eating habits is slightly more dominant among females.^[28]^ A recent study in the United Arab Emirates examined eating behavior among female university students and found that a larger eating quantity significantly increases the odds of being overweight and obese.^[29]^ Moreover, a quick eating speed is associated with a significant increase in the odds of obesity.^[29]^ A healthy diet prevents the individual from contracting many diseases such as high blood pressure, diabetes, immune diseases, heart diseases, psychological diseases, and others.^[30]^ In this study, we examined eating behaviors and patterns among adults’ Jordanian females and what are their opinions toward the trends of diets.

## 2. Materials and methods

### 2.1. Study design and settings

Between April 20, 2025 and May 31, 2025, a cross-sectional study was conducted utilizing an online survey tool in Jordan to examine female adults eating behavior.

### 2.2. Sampling procedure

The research sample was collected using convenience sampling technique. This type of sampling is a form of nonprobability sampling. This study comprised eligible females who met the inclusion criteria and were available and willing to participate. On the first page of the questionnaire, a consent form was displayed, and participants were advised that they could continue or leave. Participants were made aware of the significance of their participation by providing them with a clear statement of the study’s aims. In the research invitation letter, the inclusion criteria were specified.

### 2.3. Study population

We solicited participation from adults’ females who are currently living in Jordan. There were no restrictions on females’ socioeconomic status, body weight, or any other demographic characteristic. Males were excluded from this study.

### 2.4. Study tool

This study examined females eating behavior using the Arabic version of a previously validated questionnaire named Adult Eating Behavior Questionnaire (AEBQ).^[25]^ The questionnaire gathered information regarding participants demographics including age, current weight status, marital status, occupation, income, number of family member, and comorbidities. The second section of the questionnaire examined food approach (hunger, EOE, and enjoyment of food [EF]) and food avoidance (satiety response [SR], food fussiness [FF], and SE). The internal consistency of the 27-items AEBQ was assessed using Cronbach’s alpha. The overall scale demonstrated good reliability with Cronbach’s alpha of 0.81, indicating a strong internal consistency.

### 2.5. Piloting of the questionnaire tool

Two clinical pharmacists assessed and validated the questionnaire instrument. They were questioned about the clarity and comprehensibility of the queries, as well as their face validity and whether any were difficult to understand. Furthermore, they were inquired as to whether any of the inquiries had caused them any offense or distress. They stated that the questionnaire was straightforward to understand and complete. Furthermore, a pilot study was conducted with a limited number of females to evaluate the questionnaire’s lucidity before it was implemented on a larger scale. The results confirmed that the questionnaire is uncomplicated.

### 2.6. Ethical approval

The research ethics committee at Isra University, Amman, Jordan, approved the study protocol (SREC/25/04/138). Informed consent was obtained from the study participants prior to study commencement. This study was conducted in accordance with the World Medical Association Declaration of Helsinki.

### 2.7. Sample size

Using a confidence interval of 95%, a standard deviation of 0.5, and a margin of error of 5%. The required sample size was 385 participants.

### 2.8. Data analysis

Descriptive statistics were used to summarize the characteristics of the study participants, including frequencies and percentages for categorical variables. The continuous data was expressed by mean and standard deviation. The Kolmogorov–Smirnov test was applied to assess the normality of continuous variables. For group comparisons, independent sample *t*-tests and 1-way ANOVA were employed, Tukey-post hoc test was applied for multiple comparisons. Additionally, logistic regression analysis was conducted to predict significant factors influencing the food approach behaviors and avoidance behaviors after categorizing the scale into 2 groups, based on the mean score of 45 and 38, respectively. All data analysis was conducted utilizing SPSS software, version 29. A *P* value < .05 considered as significant

## 3. Results

A total of 702 females were involved in this study. The majority of participants were aged 18 to 23 years (579, 82.5%), single (622, 88.6%), and students (511, 72.8%). Most reported a normal weight (356, 50.7%) and had no comorbidities (659, 93.9%). Regarding monthly income, most females (333, 47.4%) reported earning between below 500 Jordanian dinar, and over half (385, 54.8%) belonged to families with 4 to 6 members. More details about demographic characteristics are provided in Table 1.

**Table 1:** Demographic characteristics of the participants.

Demographic characteristics	N	%
Age (yr)		
18–23	579	82.5%
24–29	101	14.4%
30–34	13	1.9%
35–39	2	0.3%
40–44	2	0.3%
45–50	3	0.4%
50 year and older	2	0.3%
Current weight status		
Underweight	120	17.1%
Normal	356	50.7%
Overweight	197	28.1%
Obese	29	4.1%
Marital status		
Single	622	88.6%
Married	75	10.7%
Divorced	5	0.7%
Occupation		
Not working	117	16.7%
Student	511	72.8%
Medical field	20	2.8%
Not medical field	53	7.5%
Retired	1	0.1%
Income (JOD)		
Below 500	333	47.4%
501–1000	243	34.6%
1001–1500	61	8.7%
1501 and above	65	9.3%
Number of family member		
1–3	82	11.7%
4–6	385	54.8%
7–10	228	32.5%
10 and more	7	1.0%
Comorbidities		
No	659	93.9%
Yes	43	6.1%

JOD = Jordanian dinar.

The mean food approach subscale score was 44.8 ± 10.3 (which is equivalent to 59.7% of the maximum attainable score), demonstrating more positive attitude towards approaching foods. Among the food approach subscales, “Enjoyment of Food” showed the highest mean score (21.23 ± 4.12), corresponding to 70.7% of the maximum possible score, while “Emotional Over-Eating” had the lowest mean score at 12.64 ± 5.6 (50.5%). The mean food avoidance subscale score was 38.1 ± 6.2 (which is equivalent to 63.5% of the maximum attainable score), demonstrating more positive attitude towards foods avoidance. In the food avoidance subscales, “Food Fussiness” recorded the highest mean score (14.34 ± 2.36; 71.7%), while “slowness in Eating” had the lowest mean score at 11.55 ± 3.87 (57.7%), Table 2.

**Table 2:** Mean AEBQ subscale score.

Scale	Number of items	Mean score (standard deviation)	% of maximum score
Food approach subscale	15	44.80 (10.30)	59.7
Hunger (H)	4	10.92 (3.25)	54.6
Emotional overeating (EOE)	5	12.64 (5.67)	50.5
Enjoyment of food (EF)	6	21.23 (4.12)	70.7
Food avoidance subscale	12	38.10 (6.20)	63.5
Satiety response (SR)	4	12.00 (3.24)	60.0
Food fussiness (FF)	4	14.34 (2.36)	71.7
Slowness in eating (SE)	4	11.55 (3.87)	57.7
Eating Behavior Questionnaire-Arabic (AEBQ)	27	81.20 (13.08)	60.1

AEBQ = Adult Eating Behavior Questionnaire, EF = enjoyment of food, EOE = emotional overeating, FF = food fussiness, H = Hunger, SE = slowness in eating, SR = satiety response.

Table 3 presents AEBQ subscales score stratified by sociodemographic variables. Statistically significant differences were found in FF scores by age group, with the 35 to 39 age group showing the highest mean score (18.50 ± 3.54, *P* = .001). Regarding weight status, significant differences were observed in EF (*P* < .0001), FF (*P* = .02), EOE (*P* < .001), and SR (*P* < .001), with obese individuals scoring highest in EF (23.24 ± 3.56), FR (13.93 ± 2.14), EOE (16.55 ± 5.68), and SR (10.24 ± 2.84).

**Table 3:** Eating behaviors subscales and scale by sociodemographic factors.

Sociodemographic variables	EF	*P*-value	FF	*P*-value	EOE	*P*-value	H	*P*-value	SE	*P*-value	SR	*P*-value	Total	*P*-value
Age														
18–23	21.34 ± 4.15	.32	14.45 ± 2.32	.00	12.84 ± 5.70	.29	10.95 ± 3.25	.81	11.61 ± 3.94	.22	12.26 ± 3.10	.00	83.45 ± 11.38	.28
24–29	20.86 ± 4.19		14.00 ± 2.48		12.18 ± 5.50		10.64 ± 3.22		12.19 ± 4.14		11.50 ± 3.42		81.38 ± 11.87	
30–34	22.23 ± 4.38		12.38 ± 2.79		13.77 ± 5.45		11.08 ± 2.78		10.69 ± 3.73		9.54 ± 4.29		79.69 ± 12.34	
35–39	17.00 ± 1.41		18.50 ± 3.54		7.50 ± 3.54		8.00 ± 1.41		11.50 ± 0.71		12.00 ± 1.41		74.50 ± 4.95	
40–44	17.50 ± 2.12		17.00 ± 1.41		5.00 ± 0.00		10.50 ± 2.12		13.00 ± 4.24		17.50 ± 3.54		80.50 ± 9.19	
45–50	23.33 ± 4.51		13.00 ± 2.65		11.00 ± 5.29		9.67 ± 2.08		8.00 ± 1.00		10.00 ± 3.61		75.00 ± 8.19	
50 and above	19.00 ± 2.83		13.50 ± 2.12		12.50 ± 3.54		11.50 ± 3.54		7.00 ± 4.24		12.50 ± 0.71		76.00 ± 9.90	
Current weight status														
Underweight	20.25 ± 4.64	.00	14.90 ± 2.40	.02	11.10 ± 5.43	.00	10.97 ± 3.12	.19	11.58 ± 4.38	.17	13.48 ± 3.34	.00	82.28 ± 10.54	.12
Normal	21.29 ± 3.84		14.36 ± 2.30		12.37 ± 5.34		10.77 ± 3.14		11.94 ± 3.67		12.31 ± 2.99		83.04 ± 11.33	
Overweight	21.53 ± 4.34		14.08 ± 2.52		13.76 ± 5.96		10.91 ± 3.43		11.16 ± 4.21		11.20 ± 3.17		82.64 ± 12.27	
Obese	23.24 ± 3.56		13.93 ± 2.14		16.55 ± 5.68		12.10 ± 3.22		11.69 ± 3.86		10.24 ± 2.84		87.76 ± 10.53	
Marital status														
Single	21.35 ± 4.15	.30	14.39 ± 2.37	.20	12.82 ± 5.71	.28	10.90 ± 3.28	.97	11.64 ± 3.97	.01	12.15 ± 3.18	.57	83.26 ± 11.60	.13
Married	20.56 ± 4.30		14.20 ± 2.43		11.76 ± 5.23		10.87 ± 2.82		11.41 ± 3.81		11.79 ± 3.47		80.59 ± 10.30	
Divorced	21.40 ± 2.30		12.60 ± 3.05		13.80 ± 5.76		10.60 ± 1.34		16.60 ± 4.10		11.40 ± 2.88		86.40 ± 7.96	
Widowed	0.00 ± 0.00		0.00 ± 0.00		0.00 ± 0.00		0.00 ± 0.00		0.00 ± 0.00		0.00 ± 0.00		0.00 ± 0.00	
Occupation														
Nort working	20.29 ± 4.28	.05	14.37 ± 2.34	.64	11.77 ± 5.33	.04	10.97 ± 2.98	.39	12.18 ± 4.04	.05	12.44 ± 3.43	.67	82.02 ± 11.72	.02
Student	21.53 ± 4.06		14.41 ± 2.33		12.99 ± 5.65		10.98 ± 3.25		11.65 ± 3.88		12.06 ± 3.09		83.61 ± 11.13	
Medical field	21.30 ± 5.17		13.95 ± 2.74		14.80 ± 6.01		10.70 ± 4.16		11.70 ± 4.46		12.30 ± 3.93		84.75 ± 15.60	
Not medical field	20.79 ± 4.15		14.08 ± 2.87		11.47 ± 6.11		10.09 ± 3.17		10.62 ± 4.21		11.72 ± 3.51		78.77 ± 11.49	
Retired	21.00 ± 0.00		12.00 ± 0.00		10.00 ± 0.00		9.00 ± 0.00		4.00 ± 0.00		13.00 ± 0.00		69.00 ± 0.00	
Income														
0–500	21.11 ± 4.22	.37	14.35 ± 2.19	.80	13.17 ± 5.80	.08	10.97 ± 3.25	.63	11.63 ± 3.89	.20	12.05 ± 3.30	.28	83.28 ± 12.14	.69
501–1000	21.16 ± 4.13		14.44 ± 2.46		12.28 ± 5.47		10.98 ± 3.13		11.40 ± 3.82		12.10 ± 3.06		82.35 ± 11.31	
1001–1500	21.95 ± 3.42		14.33 ± 2.90		11.52 ± 5.27		10.56 ± 3.22		11.77 ± 4.35		12.82 ± 3.16		82.95 ± 8.84	
1501 and above	21.75 ± 4.51		14.11 ± 2.57		13.18 ± 5.87		10.55 ± 3.50		12.58 ± 4.46		11.78 ± 3.28		83.97 ± 10.72	
Number of family member														
1–3	20.45 ± 4.32	.00	14.35 ± 2.37	.66	12.04 ± 5.34	.48	10.52 ± 3.12	.11	12.11 ± 4.06	.01	12.34 ± 3.24	.80	81.82 ± 11.25	.02
4–6	20.99 ± 4.17		14.44 ± 2.48		12.63 ± 5.69		10.75 ± 3.18		11.19 ± 3.95		12.15 ± 3.28		82.15 ± 11.52	
7–10	22.08 ± 3.94		14.21 ± 2.24		13.12 ± 5.71		11.30 ± 3.35		12.25 ± 3.90		11.96 ± 3.10		84.93 ± 11.27	
10 and above	19.00 ± 4.62		14.86 ± 1.77		12.29 ± 6.52		10.14 ± 1.46		12.29 ± 3.30		11.86 ± 2.41		80.43 ± 11.79	
Comorbidities														
No	21.25 ± 4.11	.77	14.34 ± 2.37	.57	12.65 ± 5.60	.20	10.88 ± 3.21	.64	11.68 ± 3.99	.45	12.14 ± 3.23	.38	82.94 ± 11.35	.67
Yes	21.44 ± 4.82		14.56 ± 2.69		13.79 ± 6.55		11.12 ± 3.45		11.21 ± 3.60		11.70 ± 2.75		83.81 ± 13.19	

EF = enjoyment of food, EOE = emotional overeating, FF = food fussiness, H = Hunger, SE = slowness in eating, SR = satiety response.

### 3.1. Factors associated with food approach and food avoidance eating behavior

Among the significant findings, overweight and obese individuals had higher odds of food approach behaviors compared to underweight individuals (OR = 1.77, 95% CI: 1.09–2.88, *P* = .02, OR = 3.40, 95% CI: 1.41–8.19, *P* = .006), respectively. Conversely, overweight and obese participants had significantly lower odds of food avoidance behaviors (OR = 0.38, 95% CI: 0.23–0.62, *P* < .001, OR = 0.27, 95% CI: 0.11–0.67, *P* = .00), respectively. Additionally, participants working in nonmedical fields had lower odds of food avoidance behaviors compared to those not working (OR = 0.36, 95% CI: 0.16–0.78, *P* = .01), Table 4.

**Table 4:** Factors associated with food approach and food avoidance eating behaviour.

	Food approach		Food avoidance	
	OR (95% CI)	*P* value	OR (95% CI)	*P* value
Age (yr)				
18–23	Reference group			
24–29	1.15 (0.65–2.05)	.627	1.34 (0.76–2.38)	.315
30–34	2.49 (0.68–9.15)	.171	0.93 (0.24–3.61)	.913
35–39	0.00 (0.00–0.00)	.999	6,91,25,92,876.35 (0.00–0.00)	.999
40–44	0.00 (0.00–0.00)	.999	2,03,50,95,642.28 (0.00–0.00)	.999
45–50	0.71 (0.05–9.27)	.797	0.00 (0.00–0.00)	.999
Current weight status				
Underweight	Reference group			
Normal	1.33 (0.86–2.05)	.206	0.68 (0.44–1.05)	.083
Overweight	1.77 (1.09–2.88)	.020	0.38 (0.23–0.62)	.000
Obese	3.40 (1.41–8.19)	.006	0.27 (0.11–0.67)	.005
Marital status				
Single	Reference group			
Married	0.99 (0.45–2.15)	.971	0.48 (0.22–1.05)	.067
Divorced	4.67 (0.43–50.79)	.205	98,00,63,839.02 (0.00–0.00)	.999
Occupation				
Not working	Reference group			
Student	1.56 (0.92–2.64)	.102	0.80 (0.47–1.35)	.400
Medical field	2.41 (0.84–6.91)	.101	1.38 (0.48–3.96)	.544
Not medical field	0.57 (0.26–1.27)	.173	0.36 (0.16–0.78)	.010
Retired	0.00 (0.00–0.00)	.999	0.71 (0.00–0.00)	.000
Income				
0–500	Reference group			
501–1000	0.74 (0.52–1.04)	.085	1.00 (0.71–1.42)	.992
1001–1500	0.58 (0.32–1.04)	.069	1.64 (0.92–2.94)	.096
1501 and above	0.96 (0.56–1.67)	.896	1.34 (0.77–2.34)	.299
Number of family members				
1–3	Reference group			
4–6	1.10 (0.60–2.01)	.766	0.54 (0.29–1.00)	.050
7–10	1.50 (0.78–2.86)	.224	0.56 (0.29–1.09)	.089
10 and above	0.55 (0.09–3.23)	.507	1.53 (0.26–9.22)	.640
Comorbidities				
No	Reference group			
Yes	1.21 (0.63–2.32)	.568	0.84 (0.43–1.64)	.602

CI = confidence interval, OR = odds ratio.

## 4. Discussion

This study aims to know the eating behaviors among female respondents in Jordan by using the AEBQ tool which contain food approach sub scale to know the trend toward food as it measures certain things such as enjoyment in food, eating in response to the environment, eating in response to negative feelings, or avoiding food altogether.^[31]^

In our study, the mean food approach subscale score was 44.8 ± 10.3 (which is equivalent to 59.7% of the maximum attainable score), demonstrating more positive attitude towards approaching foods. Among the food approach subscales, “Enjoyment of Food” showed the highest mean score (21.23 ± 4.12), corresponding to 70.7% of the maximum possible score, while “Emotional Over-Eating” had the lowest mean score at 12.64 ± 5.6 (50.5%). Enjoyment of food mean that the people experience some happiness or satisfaction when they eat food, while EOE is eating food in response to negative or positive emotions and feelings.^[31]^ This result from the current study was consistent with a study on young adults to measure their eating habits. It was proven that a large number of these young people tend to feel satisfied while eating food and a small number of them tend to be overeating when they feel bad emotions, as the average score was very low in the overeating category among the participants.^[32]^ Similarly, the same results were found in a study that included participants whose eating habits were followed from childhood to the age of twenty-two years.^[33]^ Contrary to this result, it was found that consumption of food in response to negative feelings widespread among women. It was found that women tend to overeat more when they feel these feelings, such as anxiety, emotional distress, or when they feel out of mood.^[34,35]^

Also, our study found that the mean food avoidance subscale score was 38.1 ± 6.2 (which is equivalent to 63.5% of the maximum attainable score), demonstrating more positive attitude towards foods avoidance. In the food avoidance subscales, “Food Fussiness” recorded the highest mean score (14.34 ± 2.36; 71.7%), while “slowness in Eating” had the lowest mean score at 11.55 ± 3.87 (57.7%). The term food avoidance means that individuals are not interesting while they eat food or may be the individual tend to decrease the amount of meal intake. The other terms which are the FF means refusing to try other meals that are not familiar to the individual and SE means that the person tend to spend more time in eating than normal.^[31]^ In 2 studies most of them were women, the first was on adults from Poland and Portugal and the second was from Poland, the results of both studies were similar, as the participants in this study had the following order of food-related behaviors: eating quickly was the highest, followed by avoiding food, which came in at medium levels, and then eating slowly was the lowest measure, which made our results in agreement with them.^[36,37]^

In our study, overweighted and obese individuals had higher odds of food approach behaviors compared to underweight individuals (OR = 1.77, 95% CI: 1.09–2.88, *P* = .02, OR = 3.40, 95% CI: 1.41–8.19, *P* = .006), respectively. This was aligned with a study that aimed to understand the relationship between eating patterns and body mass index (BMI), it was noted that women who suffer from obesity tend to have some eating behaviors more than women with a normal BMI, as they tend to eat when they feel some emotions or another type of eating pattern.^[38]^ On the contrary, there is a study that proved that women who suffer from a high BMI were much more committed to healthy eating than women with a normal weight.^[39]^ Conversely, overweight and obese participants had significantly lower odds of food avoidance behaviors (OR = 0.38, 95% CI: 0.23–0.62, *P* < .001, OR = 0.27, 95% CI: 0.11–0.67, *P* = .00), respectively. This can be confirmed by a study that aim to determine the relationship between women’s high BMI and food avoidance. Because women’s eating behaviors are complex, sometimes a woman may feel some negative feelings that she can release through eating. This study has proven that women are less likely to avoid food when they go through some negative circumstances.^[40]^ Additionally, participants working in nonmedical fields had lower odds of food avoidance behaviors compared to those not working (OR = 0.36, 95% CI: 0.16–0.78, *P* = .01). Previous studies were conducted to determine the link between employment status and eating behavior, it was noted that working women tend to consume less healthy food than nonworking women. For instance, there was a study in the United States of America that proved this information, such that the consumption of healthy food among nonworking women was more than that of working women.^[41]^

This study has limitations. The cross-sectional study design is not able to examine causality across independent and dependent variables. The generalizability of the study findings might be restricted due to the use of online survey study design. Self-administered survey study design is prone to reporting bias and recall bias. Therefore, the study findings should be interpreted carefully.

## 5. Conclusion

Jordanian females in this study exhibited a generally positive attitude toward both food approach and avoidance behaviors, with “Food Fussiness” and “Enjoyment of Food” being the most prominent characteristics within each domain. It is important to note that overweight and obese females exhibited significantly higher food approach behaviors and lower food avoidance tendencies, which suggests a behavioral pattern that may contribute to surplus weight. These results emphasize the intricate relationship between sociodemographic factors such as body weight, and dietary behaviors. Future research should investigate longitudinal patterns. Furthermore, qualitative research has the potential to enhance comprehension of the psychological and cultural factors that underlie these behaviors.

## Acknowledgments

We would like to acknowledge Princess Nourah bint Abdulrahman University Researchers Supporting Project number (PNURSP2025R483), Princess Nourah bint Abdulrahman University, Riyadh, Saudi Arabia.

## Author contributions

**Conceptualization:** Kanar Sweiss.

**Data curation:** Kanar Sweiss, Abdallah Y. Naser.

**Formal analysis:** Abdallah Y. Naser.

**Funding acquisition:** Kanar Sweiss.

**Investigation:** Kanar Sweiss, Abdallah Y. Naser, Alaa A. Alsharif.

**Methodology:** Kanar Sweiss.

**Project administration:** Kanar Sweiss.

**Resources:** Kanar Sweiss, Alaa A. Alsharif.

**Software:** Abdallah Y. Naser.

**Supervision:** Kanar Sweiss.

**Validation:** Kanar Sweiss.

**Visualization:** Kanar Sweiss, Abdallah Y. Naser.

**Writing – original draft:** Kanar Sweiss, Abdallah Y. Naser.

**Writing – review & editing:** Kanar Sweiss, Abdallah Y. Naser, Alaa A. Alsharif.
